# Applying formative evaluation in the mentoring of student intern nurses in an emergency department

**DOI:** 10.3389/fpubh.2022.974281

**Published:** 2022-10-19

**Authors:** Yan-ru Zhang, Rong-fang Hu, Tian-yu Liang, Jian-bang Chen, Yang Wei, Yan-hong Xing, Yan Fang

**Affiliations:** ^1^Department of Nursing, The First Affiliated Hospital of Fujian Medical University, Fuzhou, China; ^2^Department of Nursing, The First Clinical Medical College of Fujian Medical University, Fuzhou, China; ^3^Department of Nursing, The School of Nursing, Fujian Medical University, Fuzhou, China; ^4^Department of Social Work, The School of Health, Fujian Medical University, Fuzhou, China

**Keywords:** formative evaluation, nursing in the emergency department, internship, teaching, self-directed learning ability

## Abstract

**Objective:**

To explore the effectiveness of formative evaluation in the mentoring of student nursing interns in an emergency department.

**Methods:**

A total of 144 intern nursing students in the emergency department of a tertiary care hospital in Fuzhou were selected as the study subjects from July 2020 to February 2021. Adopting quasi-experimental studies methods, the students were divided into the experiment group (*n* = 74) and the control group (*n* = 70), based on their practicing rotation times. Formative evaluation methods such as in-person interviews, clinical scenario simulations, and clinical operation skills exams were conducted in the experiment group, while traditional summative evaluation methods were adopted for the control group. At the end of the intern period, a unified examination paper on professional knowledge concerning the emergency department, a cardiopulmonary resuscitation skill assessment, and a self-rating scale of self-directed learning was employed to evaluate professional theory performance, clinical practice ability, self-directed learning ability, and academic satisfaction among the nursing students, respectively.

**Results:**

The professional theoretical performance, clinical practice ability assessment scores, academic satisfaction, and self-directed learning abilities of the nursing students were significantly higher in the experiment group compared with the control group (*P* < 0.05).

**Conclusion:**

The application of formative evaluation during the mentoring of student intern nurses in an emergency department improved their professional theoretical performance, clinical practice skills, academic satisfaction, and self-directed learning abilities.

## Introduction

As an important department for treating acute and critically ill patients, the emergency department is characterized by a fast pace and the presentation of emergency cases, an environment in which theoretical knowledge and professional technical abilities are the skills that the nursing staff who work in this department require (1, 2). Nursing internship is an important stage in the process of transforming nursing students into professional nursing staff. Accordingly, a good internship evaluation system must be adopted that can be used to assess and evaluate all the relevant aspects of intern nursing students and ensure that they understand and can master all the competencies required within an emergency department.

At present, the evaluation method adopted in nursing education in China is primarily summative evaluation; this approach is convenient in terms of its implementation (3) but presents disadvantages in the form of delivering subjective evaluation, being a single evaluation method, and having a focus on memory and test-based questions (4). The purpose of summative evaluation is to rank, to judge outcomes, and it's extroverted, for others to see. Most of its evaluation subjects are single, generally for the examiner. The form of feedback is generalized and closed, which may cause nursing students to ignore the details of internship practice. Nursing education should avoid this simplistic evaluation approach as a model of ongoing professional education, where standardization, combined with cognitive immaturity, may lead to professional practice being perceived as “a cookbook practice” (5).

Formative evaluation is the process of teaching knowledge and understanding the complexity of the integrated learning among students, a process that typically includes self-evaluation by the students themselves, student peer evaluation, and students being evaluated by their teachers; these approaches can help to highlight problems within teaching methods and, accordingly, improve teaching methods over time (6). Formative evaluation mobilizes the initiative and motivation of both the students and their teachers, provides more timely feedback, promotes student learning, assists in the rational planning of learning schedules, fosters a cooperative spirit, and improves learning efficiency (6–9). When applied to clinical practice, a formative evaluation also provides nurse educators with the opportunity to assess the competencies of students; it enables them to provide timely feedback that is more useful to students as it relates to a broad understanding of the learned knowledge and the transition to professional practice in emergency care (10).

Research on formative evaluation in the field of nursing in China has mainly focused on the curriculum teaching of undergraduate nursing students and nurses in the field of cardiovascular disease (11–13) while this approach is relatively uncommon in clinical intern teaching. Additionally, incorporating skills-based techniques, evidence-based applications, and improved learning techniques in educational programs, continuously profiling nursing curricula, and integrating multiple formative evaluation methods in nursing education to support and assist students in engaging with constructivist learning methods (14) also represents new learning directions.

This study explored the use of formative evaluation system to comprehensively evaluate the knowledge, skills, attitude and emotion of nursing students in emergency clinical practice, so as to improve the comprehensive quality of nursing students in emergency clinical practice and make them truly competent for clinical work, thereby providing a reference for the construction of a formative evaluation system for the clinical internship education of nursing students.

## Objects and methods

### Study participants

Nursing students who underwent a 4-week internship in the emergency department of a tertiary care hospital in Fuzhou from July 2020 to February 2021 were selected as the study population. Based on the adoption of quasi-experimental study methods, the intern nursing students were grouped according to their internship rotation time. The students who rotated to the emergency department in odd months were allocated to the experiment group and those who rotated to the emergency department in even months were allocated to the control group. There were 74 subjects in the experiment group (71 females and 3 males), including 35 with a bachelor's degree and 39 with a junior college background. There were 70 subjects in the control group (67 females and 3 males), including 34 with a bachelor's degree and 36 with a junior college background. The differences in gender composition ratio and educational background composition ratio between the experiment and the control groups were not statistically significant (*P* > 0.05), and the data were comparable.

### Study methods

#### Program preparation stage

The general teaching responsibility system under the leadership of the head nurse was adopted, and the “one-to-one” teaching mode for mentoring nursing practice in the emergency department was conducted by the mentors. The specific requirements for mentors were as follows: Nurses with a junior college degree or above; nurses with a job title of “nurse” or higher; physically fit and dedicated nurses; nurses with 5 years or more clinical experience in emergency nursing, with a solid knowledge base of emergency department nursing, professional clinical operation skills and rich experience in emergency practice, and who had passed the training and assessment qualifications related to hospital mentoring.

Both groups of internship nurses were taught in a one-to-one mode, with clear responsibilities provided to the teaching staff. The nursing students practiced according to the requirements of the internship program in the emergency department, as shown in Table 1.

**Table 1:** The general characteristics of the mentors.

**The general characteristics**	**Number**	**Composition ratio**
**Gender**
Male	6	14.63
Female	35	85.37
**The number of years as a mentor**
1 ≤ the number of years <5	11	26.83
5 ≤ the number of years <10	24	58.54
10 ≤ the number of years <15	1	2.44
≥15	5	12.20
**The highest educational background**
The junior college	13	31.71
Undergraduate	26	63.41
Postgraduate and above	2	4.88
**Profession**
Nurse	34	82.93
Supervisor nurse	6	14.63
Deputy chief nurse and above	1	2.44

#### Mentoring and the evaluation methods for the control group

**Figure 1:**
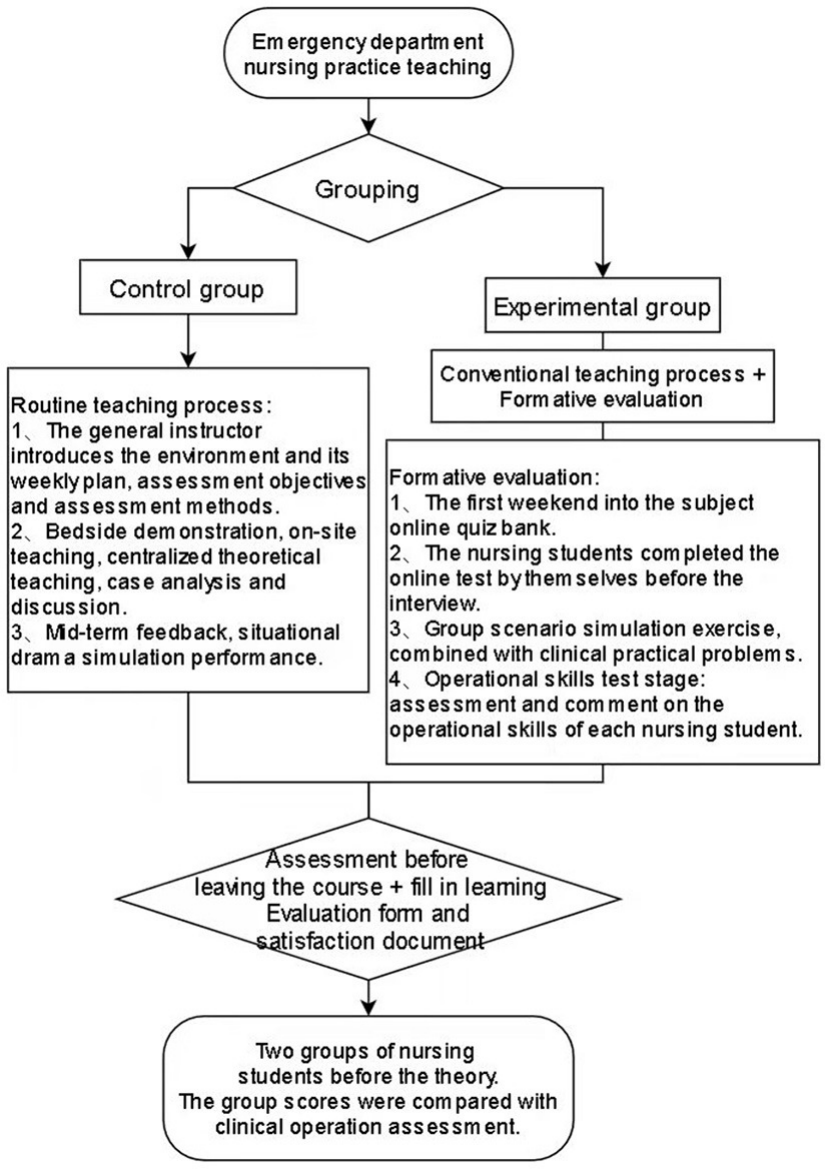
Flow chart for mentoring evaluation during the nursing practice in the emergency department (the experiment group/the control group).

Nursing students in the control group were taught according to the routine mentoring process for intern nursing students in the emergency department (Figure 1) and the details were as follows. When the nursing students rotated to the department, the chief instructor introduced the emergency department environment, a weekly mentoring plan, and clarified the final evaluation objectives and evaluation methods. Bedside demonstrations, on-site lectures, centralized theoretical lectures, case analyses, and discussions were conducted. A mid-term feedback and scenario simulation performance were conducted midway through the internship. In the final week of the internship, a concluding evaluation in the form of a theoretical examination of professional knowledge in emergency medicine and a final assessment of cardiopulmonary resuscitation (CPR) skills were conducted. Before leaving the department, the nursing students completed a self-directed learning evaluation form and a questionnaire on academic satisfaction regarding the internship process.

#### Mentoring and evaluation methods in the experiment group

For those in the experiment group, in addition to the routine mentoring process for intern nursing students in the emergency department, a formative evaluation was conducted during mentoring, which comprised four parts and accounted for 60% of their total performance in the emergency department internship (15). The details were as follows. An online question bank quiz was conducted at the end of the first week of students' admission to the department (all were multiple-choice questions, and the questions would not be repeated in the final theoretical examination paper). A focus discussion was conducted by the chief instructor in the second week of the internship, who conducted in-person interviews with each intern nurse; the intern nurse was instructed to complete the online self-assessment questions before attending the interview. Using the online platform, the chief instructor could gain an understanding of the degree of knowledge mastery for each intern nurse. During the interview, the intern nurse reported on their daily practice to the chief instructor, which included aspects like attendance, operation, and maintaining a learning diary. The chief instructor verbally enquired about emergency department nursing, and the nursing students provided answers immediately. A question-and-answer session was conducted between the nursing student and the chief instructor concerning the problems encountered during the early stage of their internship. At the end of the interview, the chief instructor summarized the performance of the nursing student during the early stage of the internship, praised and reinforced the relevant merits, and pointed out weaknesses that required improvement. Finally, the chief instructor ranked each intern nurse (a percentage score), based on the assessment score of the online question bank, the interview, and the information they provided about daily practice, which should be included in the overall evaluation (and accounted for 20%). If the student's first performance was poor and they failed (below 80 points), they could be interviewed again 1 week later and the best grade would be counted in the overall evaluation. Group scenario simulations were conducted during the third week of admission to the department, in which the nursing students performed a scenario based on the specialties of emergency department nursing, which required the analysis of clinical cases encountered during the internship, based on the clinical problems. The nursing students were encouraged to review the relevant literature and learn about new advances in emergency department care. The department provided the teaching props (e.g., dummies and manipulation tools) that were needed for the scenario simulations. The audience comprised the head nurse, the chief instructor, and the nursing student mentors. After completing a scenario, the head nurse and the chief instructor provided comments and encouraged the students to comment on the performances of their peers. Details of the performance were included in the final evaluation (accounting for 20%). An operational skills testing stage was implemented in the third week, during which each mentor assessed the students' mastery of intravenous infusion skills, and the teacher looked for problems in their operational skills and made comments while doing so. The test results were included in the final evaluation and accounted for 20% of the overall evaluation.

Finally, both groups of nursing students underwent theoretical and clinical operation skills assessment before leaving the emergency department. The theoretical knowledge examination was a professional knowledge paper of emergency medicine. The test paper included 45 multiple-choice questions with 35 single-choice questions, and 10 multiple-choice questions, and the examination duration was 45 min. The operation examination comprised a CPR skills test, the standards of which included the basic life support technology test for CPR, which encompassed an assessment of students in terms of condition observation, clinical operation skills, and humanistic care skills. The operation test duration was 6 min. The exams were conducted by the chief instructor and grades were calculated on a percentage basis. In the control group, the theoretical performance and the clinical operation performance accounted for 50% of the total evaluation score, respectively. In the experiment group, the theoretical performance and the clinical operation performance accounted for 20% of the total evaluation grade of the formative evaluation, respectively.

### Evaluation indicators

#### Exam results

The final exam results were compared between the two groups of nursing students, including their theoretical performance and clinical operation skills performance (calculated on a percentage basis). The theoretical performances and clinical operation performances accounted for 50% of the total score, respectively.

#### The self-rating scale for self-directed learning

The self-rating scale for self-directed learning (SRSSDL) was developed by Williamson (16) and translated, cross-culturally adjusted, and tested for reliability by Shen and Hu (17) from Fudan University in 2012. The SRSSDL is a self-assessment scale with 5 dimensions as follows: learning awareness, learning strategies, learning activities, learning evaluation, and interpersonal skills. Each dimension included 12 items for a total of 60 items. The Likert 5-point scale measurement was used for scoring, with the total scale scores ranging from 60 to 300 points, and higher scores indicated a more desirable self-directed learning ability for the respondent. The Chinese version of the SRSSDL was tested, and it was reported that Cronbach's alpha (α) internal coefficients were 0.97 for the SRSSDL scale as a whole and 0.87–0.90 for each of the five dimensions. The content validity was 0.96. Thus, the SRSSDL scale had good reliability and validity.

#### Nursing student internship satisfaction

The undergraduate nursing student academic satisfaction scale (UNSASS), developed by Dennision et al. (18), was adopted for the nursing student internship satisfaction (NSIS) evaluation; it was revised, translated, and modified by He and Lei (19) and included 39 entries in four dimensions, i.e., in-class teaching, clinical teaching, internship program, and support and resources within the program. The clinical teaching dimension comprised 15 entries (entries 10–24), the internship program dimension comprised 12 entries (entries 25–36), and the support and resources dimension comprised 3 entries (entries 37–39). A score of 1 point reflected “strongly disagree”, 2 points reflected “disagree”, 3 points reflected “somewhat agree”, 4 points reflected “agree”, and 5 points reflected “strongly agree”. The higher the score, the stronger the ability of the entry. The KMO value in the present study was 0.971, and the Cronbach's α coefficients were 0.92 for the total scale as a whole and ranged within the scope of 0.668–0.789 between dimensions, which indicated good reliability.

### Data collection methods

Before leaving the department in the fourth week of the internship program, the chief teaching assistant conducted a questionnaire survey using the SRSSDL and UNSASS to understand the self-directed learning ability and academic satisfaction with the internship among the nursing students in the experiment and control groups, respectively, using uniform instructional speech. In both groups, the scales were delivered by the chief teaching assistant on-site, and were completed and collected on-site.

### Statistical methods

The SPSS Statistics 20.0 software was used to conduct the data analysis. The countable data were expressed as the percentage number (*n*, %) and tested by the chi-square (X^2^) or rank-sum tests. The measurement data were expressed as means ± standard deviation ($\bar{x}$ ± s) and were tested using the *t-*test; *P* < 0.05 was considered statistically significant.

## Results

### A comparison of the exam results between the two groups of intern nursing students

The exam results were significantly higher among the nursing students in the experiment group compared with those in the control group (*P* < 0.05); the theoretical and clinical operation performances were also significantly higher among the nursing students in the experiment group compared with those in the control group, as shown in Table 2.

**Table 2:** The comparison of the rotation examination performance between the two groups of intern nursing students.

**Group**	**The control group**	**The experiment group**	***t***	***P***
The theoretical performance	87.21 ± 3.19	90.20 ± 3.11	5.689	<0.001
The operation skills performance	93.00 ± 2.33	95.35 ± 2.12	6.339	<0.001
The rotation examination performance	90.11 ± 5.66	92.80 ± 2.13	7.191	<0.001

### A comparison of the academic satisfaction between the two groups of intern nursing students

After completing the internship, the results of the NSIS related to the aspects of in-class teaching, clinical teaching, and support and resources within the program were significantly higher in the experiment group compared with the control group (*P* < 0.05), as demonstrated in Table 3.

**Table 3:** The comparison of the academic satisfaction with the internship between the two groups of intern nursing students after the internship.

**Group**	**The experiment group**	**The control group**	***t***	***P***
The in-class teaching	40.68 ± 1.895	39.26 ± 2.24	4.094	<0.001
The clinical teaching	67.28 ± 2.27	65.91 ± 2.01	3.826	<0.001
The practicing program	52.15 ± 2.54	51.17 ± 3.44	1.948	0.053
The support and resources within the program	13.61 ± 0.98	13.13 ± 1.17	2.667	0.009

### A comparison of the self-directed learning ability between the two groups of intern nursing students

Prior to the internship, the results of the SRSSDL revealed that differences in the dimensions of learning awareness, learning strategies, learning activities, and learning evaluation were not statistically significant between the nursing students in the experiment group and those in the control group (*P* < 0.05), as illustrated in Table 4.

**Table 4:** The comparison of the self-directed learning ability between the two groups of intern nursing students before the internship.

**Items**	**The experiment group**	**The control group**	***t***	***P***
The learning awareness	51.47 ± 2.67	51.29 ± 2.974	0.398	0.691
The learning strategies	51.64 ± 2.74	51.03 ± 2.95	1.280	0.203
The learning activities	51.28 ± 2.89	51.14 ± 3.36	0.269	0.788
The learning evaluation	51.55 ± 2.50	51.30 ± 2.53	0.607	0.545
The interpersonal skills	51.80 ± 2.29	51.27 ± 2.80	1.237	0.218
Total score	257.74 ± 5.84	256.03 ± 6.94	1.607	0.110

After completing the internship, the results of the SRSSDL suggested that scores in the dimensions of learning awareness, learning strategies, learning activities, and learning evaluation were significantly higher in the experiment group compared with the control group (*P* < 0.05), as shown in Table 5.

**Table 5:** The comparison of the self-directed learning ability between the two groups of intern nursing students after the internship.

**Items**	**The experiment group**	**The control group**	***t***	***P***
The learning awareness	51.62 ± 2.69	50.20 ± 3.33	2.826	0.005
The learning strategies	52.38 ± 2.63	50.77 ± 2.80	3.548	0.001
The learning activities	52.76 ± 2.66	51.74 ± 2.93	2.174	0.031
The learning evaluation	52.84 ± 2.58	51.46 ± 2.79	3.088	0.002
The interpersonal skills	52.65 ± 2.42	51.87 ± 2.52	1.8887	0.061
Total score	262.24 ± 5.65	256.04 ± 7.06	5.834	<0.001

## Discussion

### Formative evaluation helps to improve the theoretical knowledge and clinical practice skills of intern nursing students

Formative evaluation is a dynamic, complete-process assessment. Education providers should consider formative evaluation as an integral part of effective teaching and learning because of its potential to improve student performance. During the process of transformation from theoretical knowledge to practice among the nursing students, the support of formative evaluation should be implemented, which can be multifaceted, provide positive feedback, and inspire students to achieve higher levels of learning. The practice of formative evaluation includes suggestions for clinical practice, group and pair work within the classroom, peer assessment, and verbal feedback from mentors (20).

The professional training of nursing students requires direct contact with patients in clinical practice. To ensure the mastery of basic knowledge and skills, students must adhere to clinical practice in the health services field, be able to implement theoretical knowledge, and spend significant time engaged in simulated training (21). Multiple, dynamic, and timely student-targeted evaluations, guiding students during feedback sessions, as well as the identification, analysis, and solving of problems in a timely manner, based on the results of feedback and evaluation, can effectively prevent the teaching process from being detached from the actual needs, enable the prompt adjustment of teaching methods, and improve the quality of teaching (6).

In the present study, the baseline characteristics of the nursing students in the experiment and the control groups, respectively, were compared. The results showed that the differences were not statistically significant (*P* > 0.05). After completing the internship, the theoretical knowledge performance and clinical practice ability assessment scores of the nursing students in the experiment group were significantly higher than those in the control group (*P* < 0.05). These results indicated that the teaching effect of formative evaluation for internship nursing students was better compared with the summative evaluation, which was consistent with the findings of Wu et al. (22) and Gao et al. (23).

The location in the present study was the emergency department in a tertiary care hospital in Fuzhou, Fujian Province, which undertakes the clinical practice teaching of nursing students from several medical schools in Fujian Province. For those in the experiment group, in addition to the routine mentoring process for intern nursing students in the emergency department, the evaluation processes, e.g., an in-person interview with the chief instructor, clinical scenario simulations, and stage examinations, were included to provide timely evaluation and feedback on the learning attitude, emotions, theoretical performance, practice, comprehensive analysis and judgment, and learning abilities of the nursing students. This enabled mentors to better understand the mastery level of relevant knowledge among the nursing students and enabled them to adjust the teaching focus and strategy in a timely manner if needed, based on the characteristics of the students. The nursing students could also better recognize any gaps in their learning and the learning objectives and address these accordingly.

### Formative evaluation helps to improve academic satisfaction during clinical practice among intern nursing students

The results of the present study showed that the nursing students in the experimental group were significantly more satisfied with the in-class teaching, clinical teaching, as well as the support and resources available within the program compared with those in the control group at the end of the internship (*P* < 0.05). This indicated that the level of acceptance concerning formative evaluation by the intern nursing students was higher than that in the control group. For those in the experiment group, many evaluation processes were added, based on the routine mentoring process. During the experiment, contact and communication between the nursing students and the head nurse, as well as other instructors, were increased for the experiment group, which increased their sense of belonging and to some degree improved their academic satisfaction with clinical practice. During the experiment process involving formative evaluation, the process of identifying problems in stages and encouraging the nursing students to provide feedback on these problems, as well as guiding them in combining two learning styles (independent and group learning), enabled improving their learning abilities and enhanced their self-confidence concerning knowledge mastery; to some degree, their academic satisfaction with clinical practice was also increased. Formative evaluation, as a feedback tool for enhancing learning and practice, as well as understanding, can play a role in self-development and peer review (24). Introducing students to simulated environments can help to reduce the anxiety of nursing students concerning clinical practice. Assessing the achievement of learning outcomes among the students *via* formative assessment (25) can also drive the process of internship as a whole more virtuously.

The difference in the satisfaction scores among the internship program students in the present study was not statistically significant between the two groups. This was likely due to the teaching programs all including clinical teaching tasks (issued by the medical schools) to ensure the quality of clinical teaching and regulate the management of clinical teaching. The hospital strictly followed the requirements of the internship syllabus and created a thorough and detailed weekly plan. The use of reasonable, systematic, and effective teaching programs may explain why no differences were detected between the two nursing student groups concerning their academic satisfaction with the teaching programs.

### Formative evaluation helps to improve self-directed learning among intern nursing students

The results of the present study showed that the total score of self-directed learning ability among the nursing students in the experiment group was significantly higher compared with the control group at the end of the internship (*P* < 0.05). The scores related to learning awareness, learning behavior, learning strategies, and learning evaluation were also higher than those in the control group, and the differences were statistically significant (*P* < 0.05). The above results indicated that formative evaluation may help to improve the self-directed learning of nursing students when compared with a summative assessment. Qian et al. (13) explored the effect of formative evaluation in the training of cardiovascular specialist nurses, and the results showed that it could improve the independent learning abilities of students. The improved self-directed learning skills imparted by the process of formative evaluation can help to transform theoretical understanding into daily learning assessment activities. Therefore, formative evaluation can also be regarded as an evaluation system that is inseparable from the learning process (26).

The nursing specialty is a lifelong learning discipline. Improving nursing students' enthusiasm for learning and their ability to learn independently represents an important part of clinical teaching management. The formative evaluation implemented in the present study adopted the teaching needs of intern nursing students to improve the level of specialized nursing knowledge and clinical practice skills in the emergency department as a starting point. The paper focused on the timely feedback of teaching effects, as well as the guidance, motivation, and improvement of nursing students, thereby helping nursing students to dynamically regulate their learning processes and transforming them from being educated in clinical practice to being active participants in independent learning, and continuously improving their independent learning ability.

The current study showed no statistically significant differences in the interpersonal skills dimension scores related to the self-directed learning abilities between the two groups of nursing students, which may have been because the focus of nursing interns was on the patients and that during the process of nursing education of the undergraduate and junior college nursing, the care of the nursing objects and the development of interpersonal skills of nursing students were also emphasized. Furthermore, the nursing students had already gained better interpersonal skills *via* their completed school studies, which may have been a reason for the lack of differences in the interpersonal skills dimension between the two groups of students.

The innovation of this study: 1. the current domestic standard of worth promoting clinical practice nursing students evaluation system, the popularization and application of the formative assessment in recent years gradually as a way of evaluation, help to improve students' theoretical knowledge and clinical practice ability, if successful, this study can provide perfect the evaluation system of clinical practice nursing students practice base. 2. This study breaks the traditional evaluation model of emergency clinical nursing students based on terminal evaluation, and truly achieves the training and evaluation model of “ost competency as the training target”. 3. According to the application model of formative evaluation in standardized resident training in China, this study further optimized and practiced nursing students in emergency clinical practice, and the formulation process was standardized and operable.

## Conclusion

The results of the present study suggested that the application of formative evaluation for internship nursing students could improve their theoretical knowledge, clinical practice abilities, academic satisfaction with clinical teaching, and self-directed learning abilities, which could play a positive role in cultivating highly qualified and innovative clinical skills personnel with job competency.

The study has some limitations. Because the nursing students generally arrive to the department in groups of 4–6 students, to reduce the impact of confounding factors such as group interference, a single bimonthly enrollment method was adopted to reduce the subject selection bias. Additionally, the present research reflects an intervention study; accordingly, the intervention subjects and interventionists could not be blinded, and only the data analysts were blinded. Despite these shortcomings, formative evaluation may nonetheless be an effective evaluation system for mentoring nursing students during clinical practice, and future studies should further improve formative evaluation systems to achieve better results.

## Data availability statement

The original contributions presented in the study are included in the article/supplementary material, further inquiries can be directed to the corresponding author.

## Ethics statement

The studies involving human participants were reviewed and approved by The First Affiliated Hospital of Fujian Medical University. The patients/participants provided their written informed consent to participate in this study.

## Author contributions

Y-rZ and YF: conception and design of the research and critical revision of the manuscript for intellectual content. T-yL, J-bC, and Y-hX: acquisition of data. Y-rZ, R-fH, and YW: analysis and interpretation of the data. YW and Y-rZ: statistical analysis. Y-rZ, T-yL, J-bC, and Y-hX: obtaining financing. Y-rZ, YF, and R-fH: writing of the manuscript. All authors read and approved the final draft.

## Funding

This study was supported by Education and Teaching Reform Research Project of Fujian Medical University (No. J19039).

## Conflict of interest

The authors declare that the research was conducted in the absence of any commercial or financial relationships that could be construed as a potential conflict of interest.

## Publisher's note

All claims expressed in this article are solely those of the authors and do not necessarily represent those of their affiliated organizations, or those of the publisher, the editors and the reviewers. Any product that may be evaluated in this article, or claim that may be made by its manufacturer, is not guaranteed or endorsed by the publisher.
